# A comprehensive resource for integrating and displaying protein post-translational modifications

**DOI:** 10.1186/1756-0500-2-111

**Published:** 2009-06-23

**Authors:** Tzong-Yi Lee, Justin Bo-Kai Hsu, Wen-Chi Chang, Ting-Yuan Wang, Po-Chiang Hsu, Hsien-Da Huang

**Affiliations:** 1 Department of Biological Science and Technology, Institute of Bioinformatics and Systems Biology, National Chiao Tung University, Hsin-Chu 300, Taiwan; 2 Department of Biological Science and Technology, Institute of Biochemical Engineering, National Chiao Tung University, Hsin-Chu 300, Taiwan; 3 Department of Biological Science and Technology, National Chiao Tung University, Hsin-Chu 300, Taiwan; 4 Core Facility for Structural Bioinformatics, National Chiao Tung University, Hsin-Chu 300, Taiwan; 5 Department of Computer Science and Engineering, Yuan Ze University, Taoyuan 320, Taiwan; 6 Institute of Tropical Plant Science, National Cheng Kung University, Tainan 701, Taiwan

## Abstract

**Background:**

Protein Post-Translational Modification (PTM) plays an essential role in cellular control mechanisms that adjust protein physical and chemical properties, folding, conformation, stability and activity, thus also altering protein function.

**Findings:**

dbPTM (version 1.0), which was developed previously, aimed on a comprehensive collection of protein post-translational modifications. In this update version (dbPTM2.0), we developed a PTM database towards an expert system of protein post-translational modifications. The database comprehensively collects experimental and predictive protein PTM sites. In addition, dbPTM2.0 was extended to a knowledge base comprising the modified sites, solvent accessibility of substrate, protein secondary and tertiary structures, protein domains, protein intrinsic disorder region, and protein variations. Moreover, this work compiles a benchmark to construct evaluation datasets for computational study to identifying PTM sites, such as phosphorylated sites, glycosylated sites, acetylated sites and methylated sites.

**Conclusion:**

The current release not only provides the sequence-based information, but also annotates the structure-based information for protein post-translational modification. The interface is also designed to facilitate the access to the resource. This effective database is now freely accessible at .

## Background

Protein Post-Translational Modification (PTM) plays a critical role in cellular control mechanism, including phosphorylation for signal transduction, attachment of fatty acids for membrane anchoring and association, glycosylation for changing protein half-life, targeting substrates, and promoting cell-cell and cell-matrix interactions, and acetylation and methylation of histone for gene regulation [1]. Several databases collecting information about protein modifications have been established through high-throughput mass spectrometry in proteomics. UniProtKB/Swiss-Prot [2] collects many protein modification information with annotation and structure. Phospho.ELM [3], PhosphoSite [4] and Phosphorylation Site Database [5] were developed for accumulating experimentally verified phosphorylation sites. PHOSIDA [6] integrates thousands of high-confidence *in vivo *phosphorylation sites identified by mass spectrometry-based proteomics in various species. Phospho 3D [7] is a database of 3D structures of phosphorylation sites, which stores information retrieved from the phospho.ELM database and is enriched with structural information and annotations at the residue level. O-GLYCBASE [8] is a database of glycoproteins, most of which include experimentally verified O-linked glycosylation sites. UbiProt [9] stores experimental ubiquitylated proteins and ubiquitylation sites, which are implicated in protein degradation through an intracellular ATP-dependent proteolytic system. Moreover, the RESID protein modification database is a comprehensive collection of annotations and structures for protein modifications and cross-links, including pre-, co-, and post-translational modifications [10].

dbPTM [11] was developed previously to integrate several databases to accumulate known protein modifications, as well as the putative protein modifications predicted by a series of accurately computational tools [12,13]. This updated version of dbPTM was enhanced to become a knowledge base for protein post-translational modifications, which comprises a variety of new features including the modified sites, solvent accessibility of substrate, protein secondary and tertiary structures, protein domains and protein variations. We also collected literature related to PTM, protein conservations and the specificity of substrate site. Especially for protein phosphorylation, the site-specific interactions between catalytic kinases and substrates are provided. Furthermore, a variety of prediction tools have been developed for more than ten PTM types [14], such as phosphorylation, glycosylation, acetylation, methylation, sulfation and sumoylation. This work constructed a benchmark data set for computational studies of protein post-translational modification. The benchmark data set can provide a standard for measuring the performance of prediction tools that have been presented for identifying post-translational modification sites of proteins. The web interface of dbPTM is also redesigned and enhanced to facilitate the access to the proposed resource.

## Data construction and content

**Figure 1:**
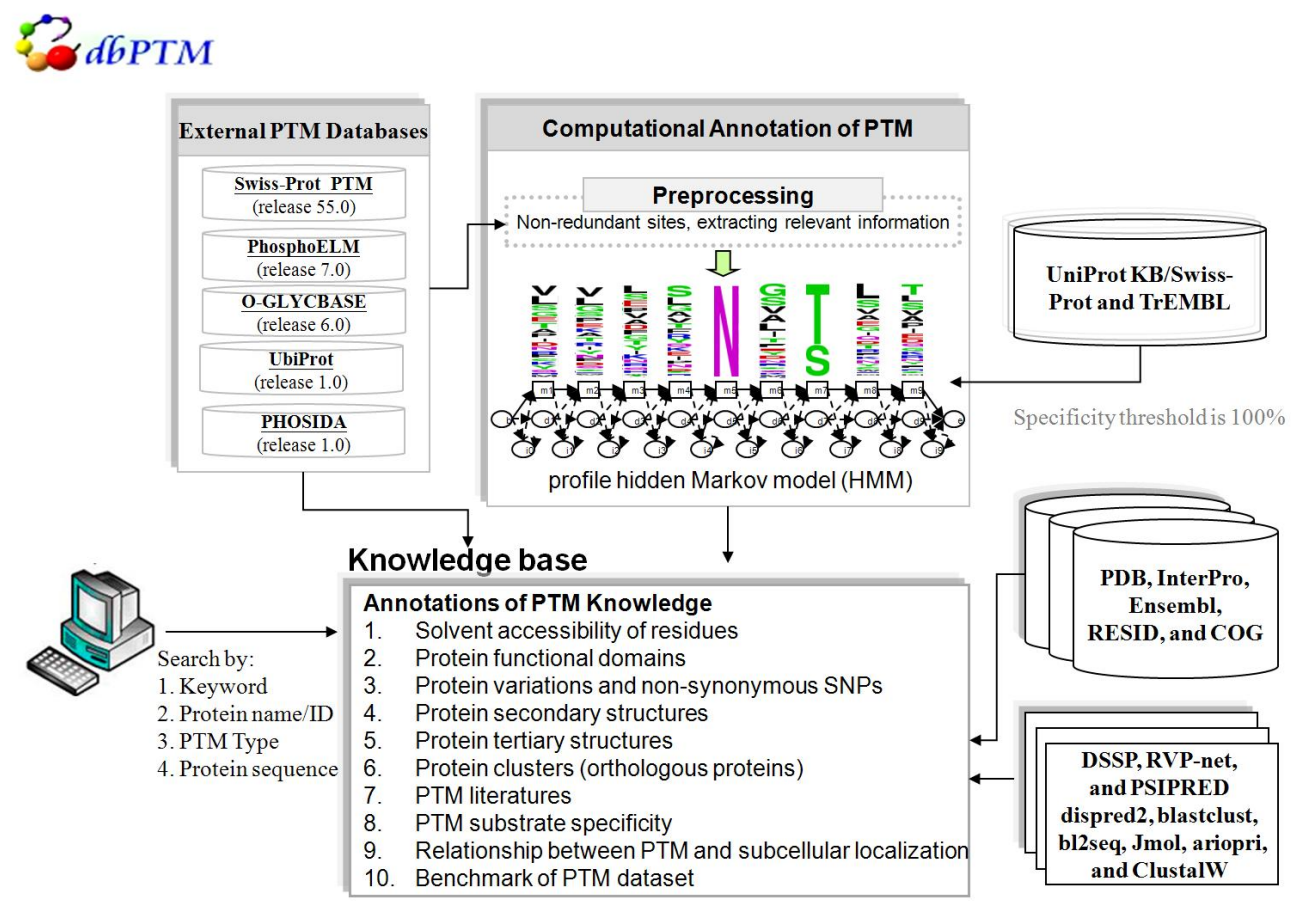
**The system architecture of the knowledge base for protein translational modification**. It comprises the three major components: integration of external experimental PTM databases, learning and prediction of 20 types of PTM, and annotations of PTM knowledge (more details in the text).

As shown in Figure 1, the system architecture of dbPTM2.0 database comprises three major components: the integration of external PTM databases, the computational identification of PTMs, and the structural and functional annotations of PTMs. We integrated five PTM databases, including UniProtKB/Swiss-Prot (release 55.0) [1], Phospho.ELM (version 7.0) [15], O-GLYCBASE (version 6.0) [8], UbiProt (version 1.0) [9] and PHOSIDA (version 1.0) [6] for obtaining experimental protein modifications. The description and data statistics of these databases are briefly given in Table S1 (see Additional file 1 – Table S1). Additionally, Human Protein Reference Database (HPRD) [16], which compiles invaluable information relevant to functions and PTMs of human proteins in health and disease, was also integrated.

In the part of computational identification of PTMs, KinasePhos-like method [11-13,17] was applied for identifying 20 types of PTM, which contain at least 30 experimentally verified PTM sites. The detailed processing flow of KinasePhos-like methods is displayed in Figure S1 (See Additional file 1 – Figure S1). The learned models were evaluated using *k*-fold cross validation. Table S2 (See Additional file 1 – Table S2) lists the predictive performance of these models. To reduce the number of false positive predictions, the predictive parameters were set to ensure a maximal of predictive specificity.

The statistics of the experimental PTM sites and putative PTM sites in this integral PTM database is given in Table 1. After removing the redundant PTM sites among six databases, there are totally 45833 experimental PTM sites in this update version. All experimental PTM sites are further categorized by PTM types. For instance, there are 31, 363 experimental phosphorylation sites and 2,080 experimental acetylation sites in the database. In addition to the experimental PTM sites, UniProtKB/Swiss-Prot provides putative PTM sites by using sequence similarity or evolutionary potential. Moreover, KinasePhos-like methods [11-13,17] were adopted to construct the profile hidden Markov models (HMMs) for twenty types of PTMs. These models were applied to identify the potential PTM sites against protein sequences obtained from UniProtKB/Swiss-Prot. As given in Table 1, 2,560,047 sites for all PTM types were identified. The structural and functional annotations of protein modifications were obtained from UniProtKB/Swiss-Prot [18], InterPro [19], Protein Data Bank [20] and RESID [10] (See Additional file 1 – Table S3).

**Table 1:** The statistics of experimental PTM sites and putative PTM sites in this study.

**PTM types**	**Modified residues**	**No. of experimental sites**	**No. of putative sites from UniProtKB/Swiss-Prot**	**No. of HMM-predicted sites in dbPTM**
Phosphorylation	Serine, threonine, tyrosine, and histidine	31,363	36,080	1,815,472

N-linked Glycosylation	Asparagine and lysine	3,264	77,571	179,955

O-linked Glycosylation	Lysine, praline, serine, threonine, and tyrosine	1,896	2,558	386,545

C-linked Glycosylation	Tryptophan	53	52	4,015

Acetylation	N-terminal of some residues and side chain of lysine or cysteine	2,080	5,143	1,206

Amidation	Generally at the C-terminal of a mature active peptide after oxidative cleavage of last glycine	2,150	1,117	24,352

Hydroxylation	Generally of asparagine, aspartate, proline or lysine	1,033	1,074	9,743

Methylation	Generally of N-terminal phenylalanine, side chain of lysine, arginine, histidine, asparagine or glutamate, and C-terminal cysteine	746	2,846	18,716

Pyrrolidone Carboxylic Acid	N-terminal glutamine which has formed an internal cyclic lactam.	598	584	12,322

Gamma-Carboxyglutamic Acid	Glutamate	371	361	1,924

Farnesylation	Cysteine	61	216	5,349

Myristoylation	Glycine	108	765	10,998

N-Palmitoylation	Cysteine	33	1,279	6,554

S-Palmitoylation	Cysteine	177	2,303	21,287

Geranyl-geranylation	Cysteine	47	819	14,317

S-diacylglycerol cysteine	Cysteine	36	1,529	8,977

GPI anchoring	C-terminal asparagine, asparate, and serine	27	681	-

Deamidation	Amidated asparagine and glutamine (needs to be followed by a G)	38	26	2,022

Sulfation	Serine, threonine, and tyrosine	196	626	15,654

Sumoylation	Lysine	77	259	10,342

Ubiquitylation	Lysine	286	516	8,865

ADP-ribosylation	Arginine	3	203	-

Formylation	Of the N-terminal methionine	28	35	-

Citrullination	Arginine	27	91	-

Nitration	Tyrosine	47	5	1,432

Bromination	Tryptophan	18	3	-

FAD	O-8alpha-FAD tyrosine, Pros-8alpha-FAD histidine, S-8alpha-FAD cysteine, and Tele-8alpha-FAD histidine	12	116	-

S-nitrosylation	Cysteine	9	93	-

Others		1049	2,958	-

Total		45,833	139,909	2,560,047

## Utility and major improvements

In order to provide more effective information about protein modifications in this update version, we extended dbPTM to a knowledge base containing structural properties for PTM sites, PTM related literature, evolutionary conservation of PTM sites, subcellular localization of modified proteins and the benchmark set for computational studies. Table 2 shows the enhancement and new features supported in this study. First of all, the integrated PTM resource is more comprehensive than previous dbPTM, which enriches the PTM types, varying from 373 to 431 PTM types. To detect the potential PTM sites in UniProtKB/Swiss-Prot proteins without any PTM annotations, the KinasePhos-like method was applied to 20 PTM types. Especially in protein phosphorylation, more than 60 kinase-specific prediction models were constructed and applied to identify the phosphorylation sites with catalytic kinases.

**Table 2:** The enhanced features in this expanding PTM database (dbPTM 2.0).

**Features**	**Previous PTM database **[11]	**dbPTM 2.0**
Protein entry	UniProtKB/Swiss-Prot (release 46)	UniProtKB/Swiss-Prot (release 55)

Experimental PTM resource	UniProtKB/Swiss-Prot, Phospho.ELM, and O-GLYCBASE	UniProtKB/Swiss-Prot, Phospho.ELM, PHOSIDA, HPRD, O-GLYCBASE, and UbiProt

Computationally predicted PTMs	Phosphorylation, glycosylation, and sulfation	About 25 types of PTM (phosphorylation, glycosylation, sulfation, acetylation, methylation, sumoylation, hydroxylation, etc.)

Protein structure	Protein Data Bank (PDB)	Protein Data Bank (PDB)

PTM annotation	RESID (373 PTM annotations)	RESID (431 PTM annotations)

Structural investigation of PTM sites	-	Solvent accessibility, secondary structure and intrinsic disorder region

Kinase family annotation	-	KinBase

Protein domain	InterPro	InterPro

Protein variation	Swiss-Prot and Ensembl	Swiss-Prot and Ensembl

Site-specific PTM literature	-	Extracting the PTM-related literatures from UniProtKB/Swiss-Prot, Phospho.ELM, HPRD, O-GLYCBASE, and UbiProt

Substrate specificity	-	Amino acid frequency, solvent accessibility, secondary structure and disorder region surrounding modified sites

Evolutionary conservation of PTM sites	-	COG and ClustalW

PTM benchmark set for computational studies	-	Providing the benchmark for constructing PTM test set to compare the predictive performance of prediction tools

Relationship between PTM and subcellular localization	-	Analyzing the relationship between PTM and subcellular localization

Graphical visualization	PTM, solvent accessibility, secondary structure, protein variation, protein domain, and tertiary structure	PTM, solvent accessibility, secondary structure, protein variation, protein domain, tertiary structure, orthologous conserved regions, substrate site specificity and protein interaction network

### Structural properties of PTM sites

In order to facilitate the investigation of structural characteristics surrounding the PTM sites, protein tertiary structure obtained from Protein Data Bank [20] was graphically presented by Jmol program. For proteins with tertiary structures (5% of UniProtKB/Swiss-Prot proteins), the protein structural properties, such as solvent accessibility and secondary structure of residues, were calculated by DSSP [21]. The solvent accessibility of residues and secondary structure of residues for proteins without tertiary structures were predicted by RVP-net [22] and PSIPRED [23], respectively. The intrinsic disorder regions were provided using Disopred2 [24].

**Figure 2:**
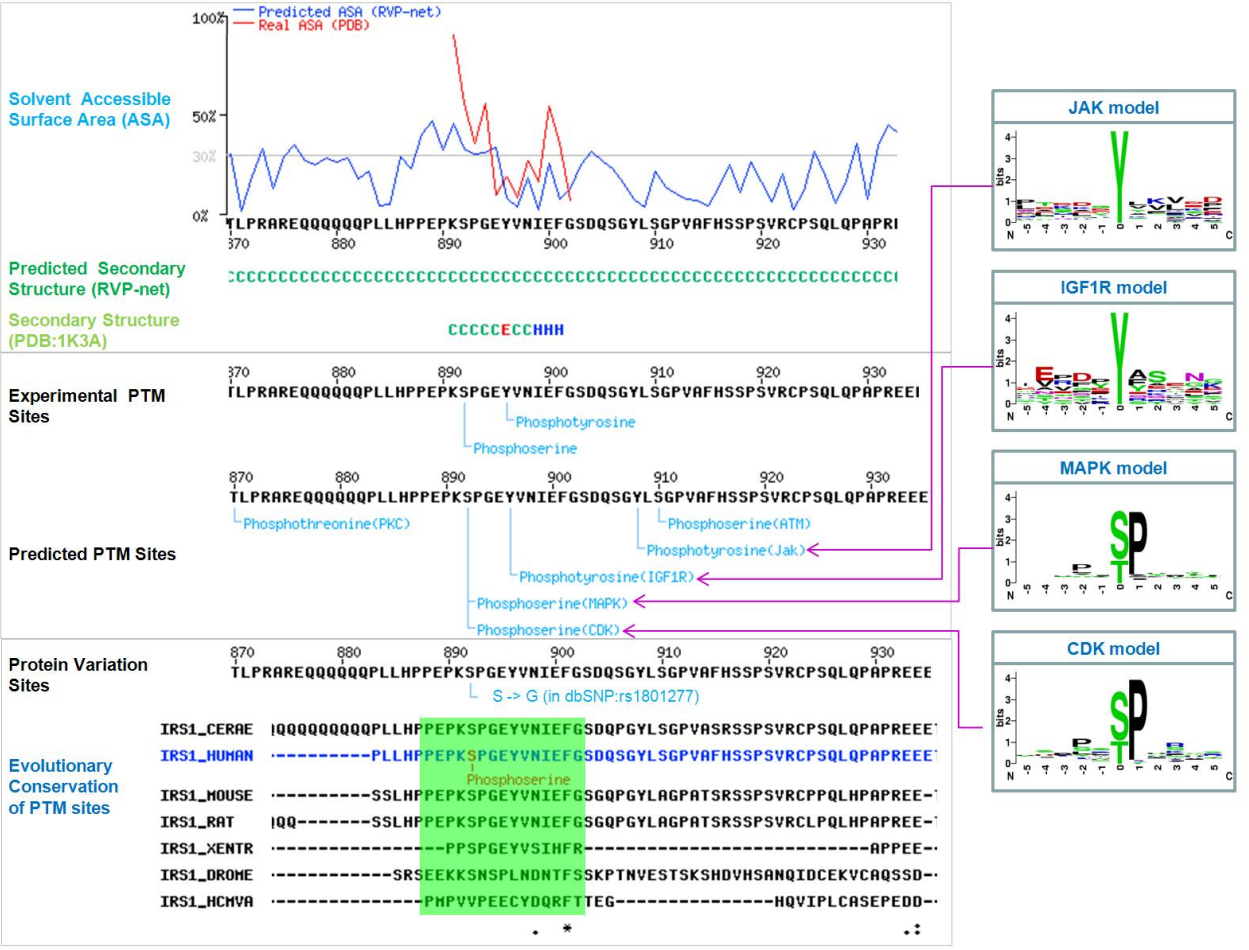
**A part of result page on the web interface**. An example of graphical presentation of PTM sites and the structural characteristics of human protein IRS1.

Figure 2 depicts an illustrative example that *Insulin Receptor Substrate 1 *(*IRS1*) of human (UniProtKB/Swiss-Prot ID: IRS1_HUMAN) can interact with *Insulin Receptor *(*INSR*) and involve in the insulin signaling pathway [25]. Three fragments of ISR1 protein have tertiary structures in PDB. Structure 1K3A the protein region from 891 AA to 902 AA. Two experimental phosphorylation sites S892 and Y896 locate in the region, and their solvent accessibility and secondary structure can be derived from the tertiary structures. The solvent accessibility and secondary structure in other protein regions without tertiary structures were calculated by the integrated programs, RVP-net and PSIPRED, respectively.

### Annotation of catalytic kinases of protein phosphorylation sites

In addition to the experimental annotations of catalytic kinases of protein phosphorylation, we applied KinasePhos-like prediction method [11-13,17] for identifying 20 types of PTM. Figure 2 gives an example that the experimental phosphorylation site S892 of *IRS1 *was predicted to be catalyzed by protein kinase MAPK and CDK with the preference of proline occurred on position -2 and +1 surrounding the phosphorylation site (position 0). Besides, Y896 is predicted to be catalyzed by kinase IGF1R, the result is consistent with previous investigation [26]. Moreover, S892 is a protein variation site, which was mapped to a non-synonymous single nucleotide polymorphism (SNP), based on the annotation obtained from dbSNP [27].

### Evolutionary conservation of PTM sites

In order to determine whether a PTM sites is conserved among orthologous protein sequences, we integrated the database of Clusters of Orthologous Groups (COGs) [28], which collected 4873 COGs in 66 unicellular genomes and 4852 clusters of eukaryotic orthologous groups (KOGs) in 7 eukaryotic genomes. ClustalW [29] program was adopted to implement the alignment of multiple protein sequences in each cluster, and the aligned profile is provided in the resource. An experimentally verified acetyllysine located in a protein-conserved region indicates an evolutionary influence in which orthologous sites in other species could be involved in the same type of PTM (See Additional file 1 – Figure S2). Furthermore, as the example shown in Figure 2, two experimentally verified phosphorylation sites are conserved.

### PTM benchmark data set for bioinformatics study

Due to the high-throughput of mass spectrometry in proteomics, the experimental substrate sequences of more than ten PTM types, such as phosphorylation, glycosylation, acetylation, methylation, sulfation and sumoylation, were investigated and used for developing the prediction tools [14]. To understand the predictive performance of these tools previously developed, it is crucial to have a common standard for evaluating the predictive performance among various prediction tools. Therefore, we constructed a benchmark, which comprise the experimental substrate sequences for each PTM type.

The process to compile the evaluation sets is described in Figure S3 (See Additional file 1 – Figure S3), based on criteria developed by Chen *et al*. [30]. To remove the redundancy, the protein sequences containing the same type of PTM sites are grouped by a threshold of 30% identity by BLASTCLUST [31]. If the identity of two protein sequences is greater than 30%, we re-aligned the fragment sequences of the substrates by BL2SEQ. If the fragment sequences of two substrates with the same location are identical, only one of the substrate was included in the benchmark data set. Therefore, twenty PTM types containing more than 30 experimental sites were complied in the benchmark data set.

### Enhanced web interface

A user-friendly web interface is provided for simple searching, browsing, and downloading of protein PTM data. In addition to the database query by the protein name, gene name, UniProtKB/Swiss-Prot ID or accession, it allows the input of protein sequences for similarity search against UniProtKB/Swiss-Prot protein sequences (See Additional file 1 – Figure S4). To provide an overview of PTM types and their modified residues, a summary table is provided for browsing the information and the annotations about the post-translational modification types, which are referred to the UniProtKB/Swiss-Prot PTM list  and RESID [10].

**Figure 3:**
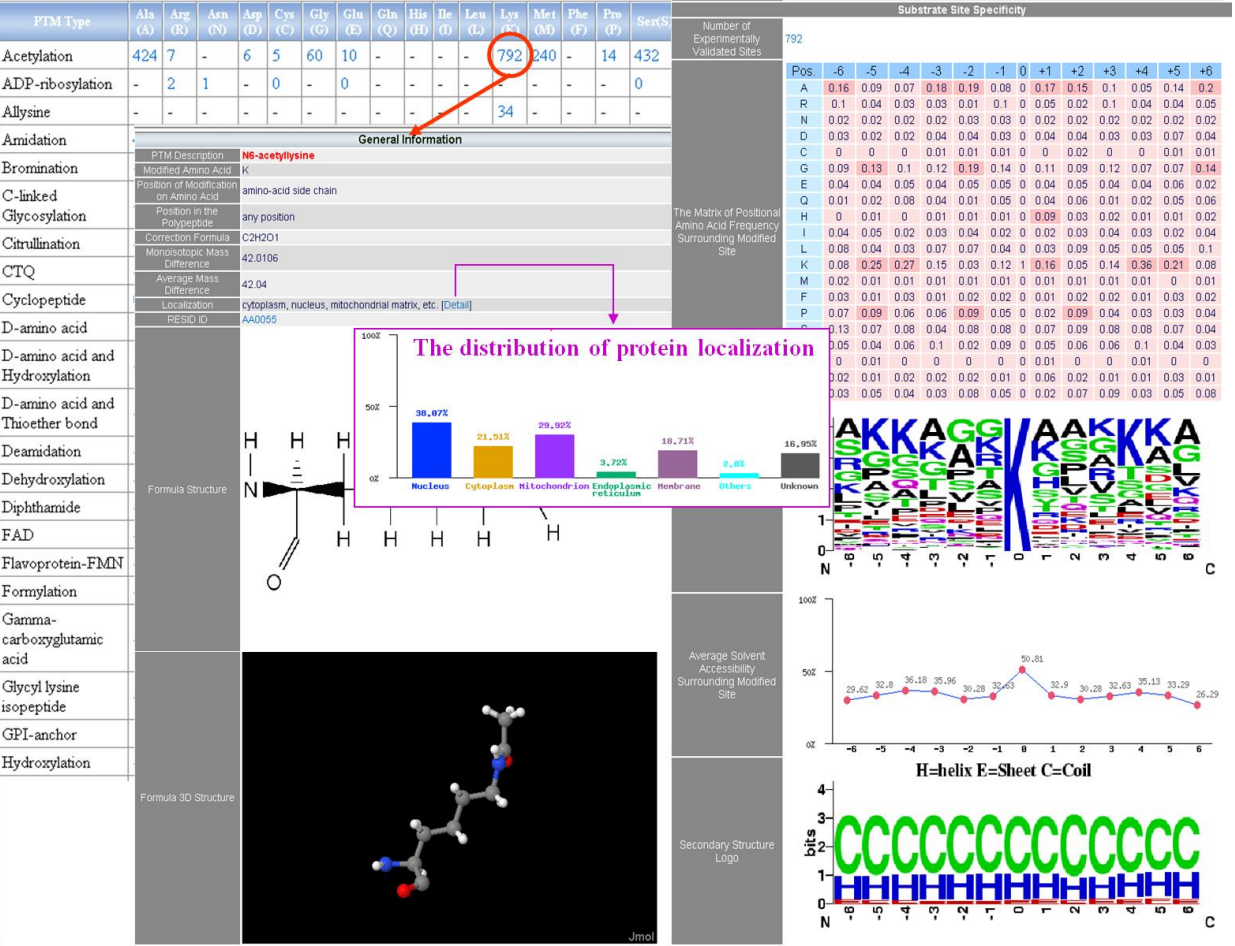
**An illustrative example to show the catalytic specificity of acetyllysine**.

Figure 3 shows an example that users can choose the acetylation of lysine (K) to obtain more detailed information such as the position of modified amino acid, the location of the modification in protein sequence, the modified chemical formula, the mass difference, and the substrate site specificity, which is the preference of amino acids surrounding the modification sites. Furthermore, the structural information, such as solvent accessibility and secondary structure surrounding the modified sites, are provided. All the experimental PTM sites and putative PTM sites can be downloaded from the web interface.

## Conclusion

The proposed server enables both wet-lab biologists and bioinformatics researchers to easily explore the information about protein post-translational modifications. This study not only accumulates the experimentally verified PTM sites with relevant literature references, but also computationally annotates twenty types of PTM sites against UniProtKB/Swiss-Prot proteins. As given in Table 2, the proposed knowledge base provides effective information of protein PTMs, including sequence conservation, subcellular localization and substrate specificity, the average solvent accessibility and the secondary structure surrounding the modified site. Moreover, we construct a PTM benchmark data set that can be adopted for computational studies in evaluating the predictive performance of various tools about determining PTM sites. Previous investigations have indicated that many protein modifications cause binding domains for specific protein-protein interaction to regulate cellular behavior [32]. All the experimental PTM sites and putative PTM sites are available and downloadable in the web interface. Prospective work of dbPTM is to integrate protein-protein interaction data.

## Availability and requirements

Project name: dbPTM 2.0: A Knowledge Base for Protein Post-Translational Modifications

ASMD project home page: 

Operating system(s): Platform-independent

Programming Language: PHP, Perl

Other requirements: a modern web browser (with CSS and JavaScript support)

Restrictions to use by non-academics: None

## List of abbreviations

PTM: Post-Translational Modification; HMMs: hidden Markov models; PDB: Protein Data Bank; SNP: single nucleotide polymorphism.

## Competing interests

The authors declare that they have no competing interests.

## Authors' contributions

HDH conceptualized the project. TYL and HDH designed and built the database. TYL, PCH and WCC performed data analysis. TYL and JBKH designed and built the interfaces. TYL, JBKH and TYW compiled a previous version of the database. HDH, TYL and WCC wrote the draft. All authors tested the database and interfaces. All authors read and approved the final manuscript.

## Supplementary Material

Additional file 1 **Supplementary figures (S1, S2, S3, and S4) and tables (S1, S2, and S3)**. The data provided 4 figures and 3 tables. The description of each figures and tables are given below. **Figure S1**. The detailed processing flow of KinasePhos-like methods. **Figure S2**. The multiple sequence alignment of orthologous conserved regions. **Figure S3**. The flowchart to remove data redundance. **Figure S4**. Example of search web pages. **Table S1**. Data statistics of the integrated resources. **Table S2**. The parameters and predictive performance of the trained models with best accuracy for each PTM type. **Table S3**. The list of integrated databases and programs. Click here for file
