# Supplementary material for: A comprehensive resource for integrating and displaying protein post-translational modifications
Source: BMC Res Notes. 2009 Jun 23;2:111. doi: 10.1186/1756-0500-2-111 (PMC2713254; doi:10.1186/1756-0500-2-111)
Supplement: Additional file 1 — Supplementary figures (S1, S2, S3, and S4) and tables (S1, S2, and S3). The data provided 4 figures and 3 tables. The description of each figures and tables are given below. Figure S1. The detailed processing flow of KinasePhos-like methods. Figure S2. The multiple sequence alignment of orthologous conserved regions. Figure S3. The flowchart to remove data redundance. Figure S4. Example of search web pages. Table S1. Data statistics of the integrated resources. Table S2. The parameters and predictive performance of the trained models with best accuracy for each PTM type. Table S3. The list of integrated databases and programs. [file 1756-0500-2-111-S1.doc]

**Additional file 1**

**FIGURES**

**Figure S1.** The detailed processing flow of KinasePhos-like methods.

**Figure S2**. The multiple sequence alignment of orthologous conserved regions.

**Figure S3**. The flowchart to remove data redundance.

**Figure S4**. Example of search web pages.

**TABLES**

**Table S1**. Data statistics of the integrated resources.

**Table S2.** The parameters and predictive performance of the trained models with best accuracy for each PTM type.

**Table S3**. The list of integrated databases and programs.

**FIGURES**


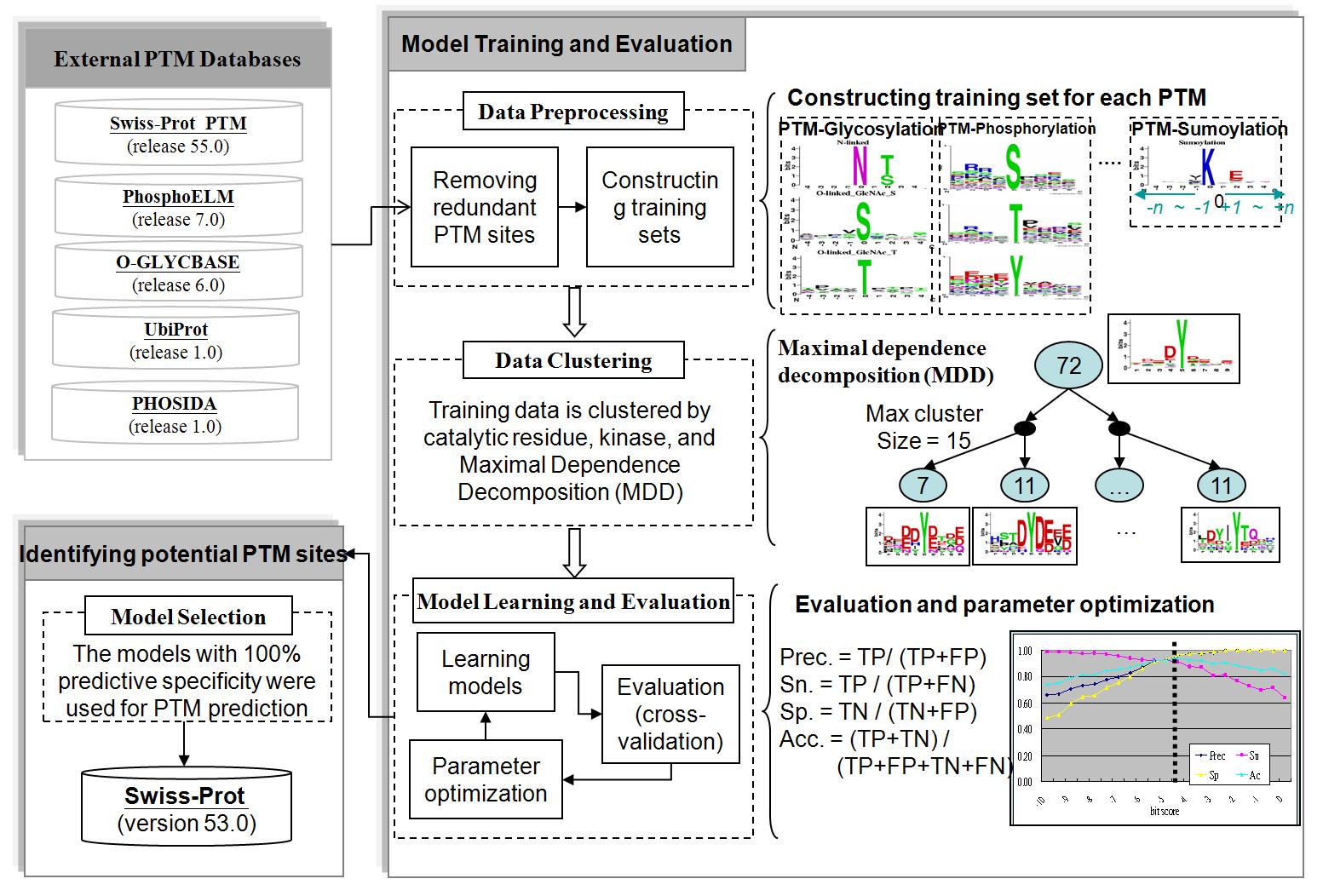


**Figure S1. The detailed processing flow of KinasePhos-like methods.** The redundant PTM sites among the four databases were removed; furthermore, about 20 types of PTM with at least 30 experimentally validated sites were used to investigate the amino acids surrounding the modified sites and train the profile HMMs. Given the window length *n*, the fragments of 2*n*+1 residues centering on PTM site (position 0) are extracted and constructed as the positive training set. The value of *n* is set to 6. However, the window lengths in several types of PTM which occurred on N-terminal or C-terminal of protein sequence are set to 0 ~ +6 or -6 ~ 0. Due to the absence of confirmed non-PTM sites, the residues that had not been annotated as PTM sites within PTM annotated proteins were chosen as a representation of general non-PTM sites (negative training set). The Maximal Dependence Decomposition (MDD) [2], which was firstly applied in the prediction of RNA splicing sites, employs statistical $\chi^{2}$-test to group a set of aligned signal sequences to moderate a large group into subgroups that capture the most significant dependencies between positions. **In each type of PTM, the profile Hidden Markov Models** (HMMs), which describes a probability distribution over a potentially infinite numbers of sequences, was adopted to train the computation models from the positive sets of the PTM site sequences aligned without gaps. Herein, we use the software package HMMER (version 2.3.2) [3] to build the models, to calibrate the models and to search the putative PTM sites against the protein sequence. Two important parameters of HMMER should be considered, bit score and expectation value (*E*-value). A search of a model with the bit score greater than the threshold *t* and the *E*-value smaller than the threshold *e* is defined as a positive prediction. We select the HMMER bit score as the criteria to define a HMM match. The threshold *t* of HMM in each type of PTM is decided by maximizing the accuracy measure during a variety of cross-validation with the bit score value range from -10 to 0. Table S2 summarizes the predictive performance of the trained models in 20 types of PTM. Finally, **we set the predictive parameters as the values when the prediction specificity is 100% and fully detect the potential PTM sites against Swiss-Prot protein sequences.**


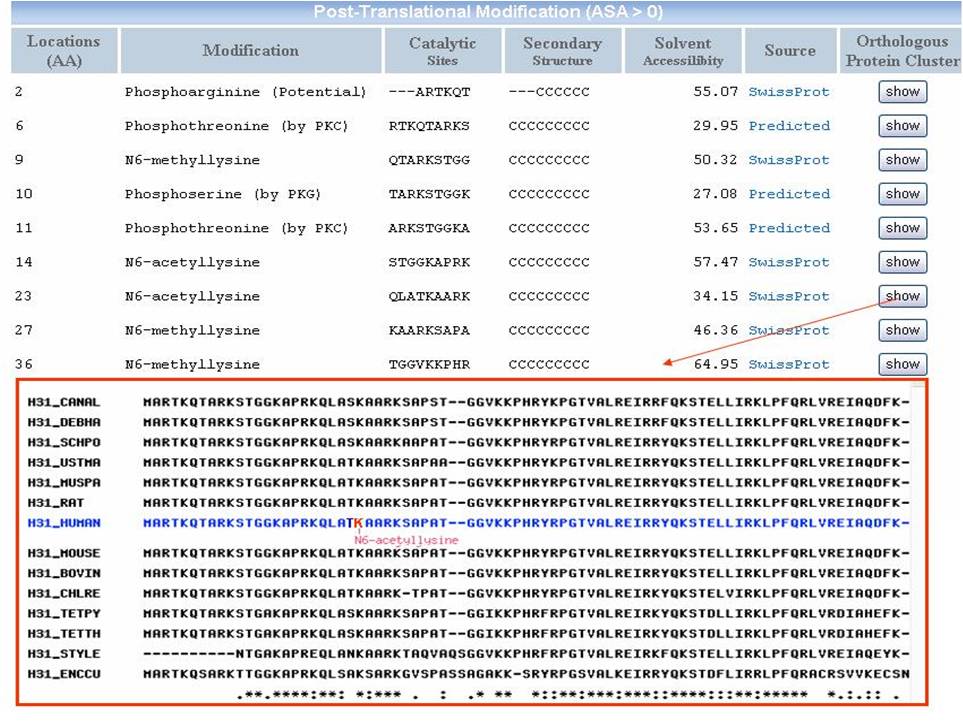


**Figure S2**. **The multiple sequence alignment of orthologous conserved regions.** Users can investigate whether or not a PTM site is located in orthologous conserved regions. The Clusters of Orthologous Groups of proteins (COGs) [4], which were delineated by comparing protein sequences encoded in complete genomes, were integrated. The COG collection currently consists of 4873 COGs in 66 genomes of unicellular organisms and 4852 clusters of eukaryotic orthologous groups (KOGs) in 7 eukaryotic genomes. Furthermore, the protein sequences in each cluster are aligned by a multiple sequence alignment tool, ClustalW [5].


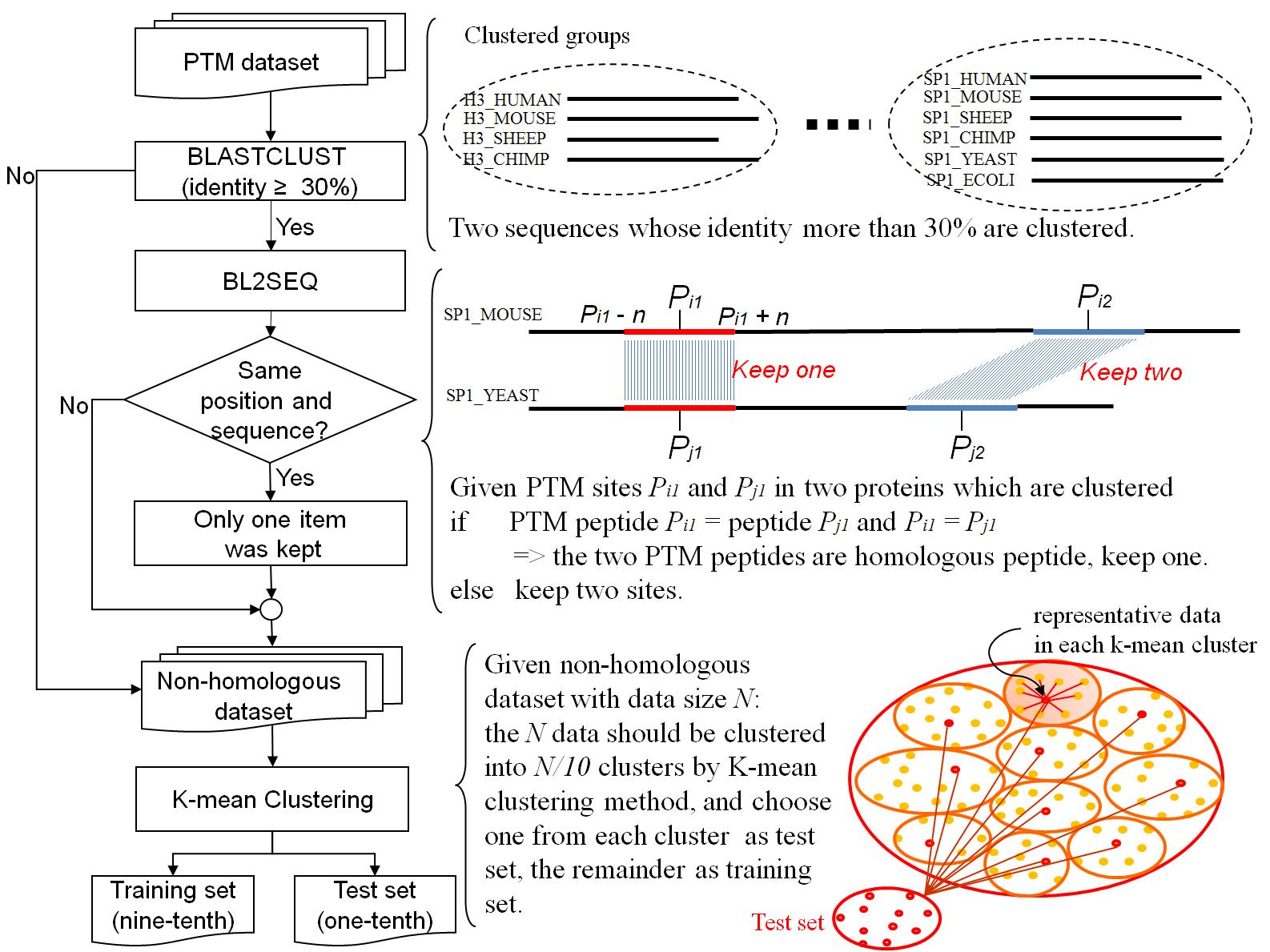


**Figure S3**. **The flowchart to remove data redundancy.** The protein sequences containing the same type of PTM sites were clustered with a threshold of 30% identity by BLASTCLUST [6]. If two protein sequences were similar with ≥30% identity, we re-aligned the fragment sequences with window length 2*n*+1 residues centering on modified sites by BL2SEQ. If two PTM fragment sequences were similar with 100% identity and when two PTM sites of the two proteins were in the corresponding positions in the alignment, only one was kept.


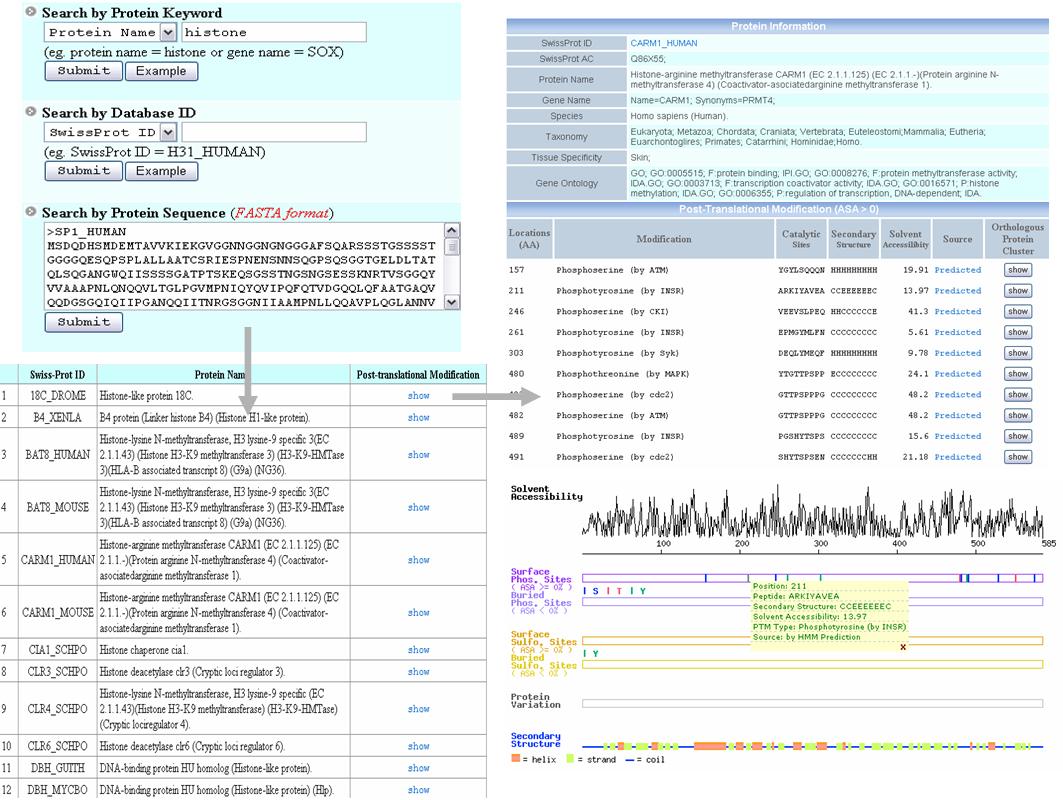


**Figure S4**. **Example of search web pages.** The proteins related to the queried word “histone” are shown in a table. Users can select a protein to view the experimental and predicted PTM sites in tabular and graphical visualizations. Furthermore, the graphical visualization reveals the post-translational modifications, the solvent accessibility of the residues, protein variations, protein secondary structures and protein functional domains.

**TABLES**

**Table S1**. **Data statistics of the integrated resources.** Six external biological databases related to protein post-translational modifications, such as UniProtKB/Swiss-Prot [7], Phospho.ELM [8], O-GLYCBASE [9], UbiProt [10], PHOSIDA [11], and HPRD [1] are integrated into the proposed knowledge base. UniProtKB/Swiss-Prot release 55.0 contributes 36618 experimental validated PTM sites within 11657 proteins, and 137915 putative PTM sites (annotated as “by similarity”, “potential” or “probable” in the ‘MOD_RES’, “CARBOHYD”, “LIPID” and “CROSSLNK” fields) within 41380 proteins. The Phospho.ELM entries store information about substrate proteins with the exact positions of residues are known to be phosphorylated by cellular kinases. 16,428 experimentally verified phosphorylation sites within 4,026 proteins were obtained from Phospho.ELM version 7.0 [12]. PHOSIDA integrates thousands of high-confidence in vivo phosphorylation sites identified by mass spectrometry-based proteomics in various species. O-GLYCBASE [9] version 6.0 provides 242 glycoproteins containing 2,765 experimentally verified O-linked, N-linked, and C-linked glycosylation sites. However, 185 glycoproteins in O-GLYCBASE are corresponded to Swiss-Prot proteins, which have 2,353 experimentally verified glycosylation sites. Especially, a novel PTM database, UbiProt, stores 417 ubiquitylated proteins which contain 165 ubiquitylation sites. In release 7.0 of HPRD, there are totally 16972 PTMs within 2830 protein entries, of 7438 PTMs are phosphorylation sites within 1774 proteins.

| **Resources** | **Version** | **Description** | **Statistics** |
| --- | --- | --- | --- |
| UniProtKB/Swiss-Prot | 55.0 | Experimental Post-Translational Modifications (PTMs) | 36,618 PTM sites within 11,657 proteins |
|  |  | Putative PTMs (annotated as “by similarity”, “potential” or “probable” in the ‘MOD_RES’, “CARBOHYD”, “LIPID” and “CROSSLNK” fields) | 137,915 PTM sites within 41,380 proteins |
| PhosphoELM | 7.0 | Experimental phosphorylation sites | 16,428 phosphorylation sites within 4,026 proteins |
| PHOSIDA | 1.0 | In vivo phosphorylation sites which was identified by mass spectrometry-based Proteomics | More than 6600 phosphorylation sites on 2244 proteins in response to EGF stimulation |
| O-GLYCBASE | 6.0 | Experimental glycosylation sites | 2,353 PTM sites within 185 glycoproteins |
| UbiProt | 1.0 | Ubiquitylated protein and ubiquitylation sites | 417 Ubiquitylated proteins and 165 ubiquitylated sites |
| HPRD | 7.0 | Experimentally validated PTM sites in human proteins | 16972 PTMs within 2830 human proteins |

**Table S2. The parameters and predictive performance of the trained models with best accuracy for each PTM type.** These parameters including window length and HMMER bit score are optimized iteratively in the process of cross-validation. (Abbrev. Prec: Precision; Sn: Sensitivity; Sp: Specificity; Acc: Accuracy)

| **PTM Types** | **Substrates** | **No. of PTM sites** | **Window length** | **HMM bit score** | **Prec** | **Sn** | **Sp** | **Acc** |
| --- | --- | --- | --- | --- | --- | --- | --- | --- |
| N-linked glycosylation | Asparagines (GlcNAc) | 3019 | -6 ~ +6 | -4.5 | 0.85 | 0.98 | 0.83 | 0.91 |
| O-linked glycosylation | Serine (GalNAc) | 212 | -6 ~ +6 | -5 | 0.80 | 0.85 | 0.79 | 0.82 |
|  | Serine (GlcNAc) | 35 | -6 ~ +6 | -6 | 0.81 | 0.71 | 0.83 | 0.77 |
|  | Serine (Man) | 79 | -6 ~ +6 | -5 | 0.88 | 0.74 | 0.90 | 0.82 |
|  | Threonine (GalNAc) | 386 | -6 ~ +6 | -4.5 | 0.81 | 0.75 | 0.82 | 0.79 |
|  | Threonine (GlcNAc) | 42 | -6 ~ +6 | -4 | 0.77 | 0.82 | 0.76 | 0.79 |
|  | Threonine (Man) | 83 | -6 ~ +6 | -7 | 0.83 | 0.88 | 0.81 | 0.85 |
|  | Lysine (Gal) | 46 | -6 ~ +6 | -5 | 1.00 | 1.00 | 1.00 | 1.00 |
| C-linked glycosylation | Tryptophane (Man) | 49 | -6 ~ +6 | -0.5 | 1.00 | 0.98 | 1.00 | 0.99 |
| Phosphorylation | Serine (kinase-specific) | 22640 | -6 ~ +6 | -5.5 | 0.88 | 0.84 | 0.88 | 0.86 |
|  | Threonine (kinase-specific) | 4982 | -6 ~ +6 | -4 | 0.91 | 0.92 | 0.91 | 0.91 |
|  | Tyrosine (kinase-specific) | 3175 | -6 ~ +6 | -5 | 0.86 | 0.81 | 0.87 | 0.84 |
|  | Histidine | 41 | -6 ~ +6 | -3 | 0.90 | 0.80 | 0.91 | 0.86 |
| Acetylation | N-acetylalanine | 403 | 0 ~ +6 | -6 | 0.64 | 0.72 | 0.60 | 0.66 |
|  | N6-acetyllysine | 292 | 0 ~ +6 | -6 | 0.77 | 0.73 | 0.79 | 0.76 |
|  | N-acetylmethionine | 199 | 0 ~ +6 | -4 | 0.83 | 0.75 | 0.85 | 0.80 |
|  | N-acetylserine | 402 | 0 ~ +6 | -4 | 0.59 | 0.84 | 0.42 | 0.63 |
|  | N-acetylthreonine | 58 | 0 ~ +6 | -6 | 0.85 | 0.53 | 0.90 | 0.71 |
| Methylation | Methylarginine | 180 | -6 ~ +6 | -1 | 0.97 | 0.78 | 0.98 | 0.88 |
|  | Methyllysine | 407 | -6 ~ +6 | 0 | 0.83 | 0.60 | 0.88 | 0.74 |
| Myristoylation | N-myristoyl glycine | 100 | -6 ~ +6 | -10 | 0.99 | 0.91 | 0.99 | 0.95 |
| Palmitoylation | N-palmitoyl csteine | 58 | -6 ~ +6 | -5 | 0.88 | 0.93 | 0.88 | 0.91 |
|  | S-palmitoyl csteine | 169 | -6 ~ +6 | -4 | 0.94 | 0.70 | 0.95 | 0.83 |
| Farnesylation | S-farnesyl cysteine | 63 | -6 ~ +6 | -4 | 0.78 | 0.89 | 0.75 | 0.82 |
| Geranyl-geranylation | S-geranylgeranyl cysteine | 52 | -6 ~ 0 | -6 | 0.69 | 0.88 | 0.61 | 0.74 |
| Hydroxylation | 4-hydroxyproline | 392 | -6 ~ +6 | -4 | 0.82 | 0.88 | 0.81 | 0.84 |
|  | 5-hydroxylysine | 83 | -6 ~ +6 | -3 | 0.97 | 0.84 | 0.98 | 0.91 |
|  | Hydroxyproline | 188 | -6 ~ +6 | -1 | 0.83 | 0.79 | 0.84 | 0.81 |
|  | 3,4-dihydroxyproline | 55 | -6 ~ +6 | -1 | 0.67 | 1.00 | 0.51 | 0.75 |
| Deamidation | Deamidated asparagin | 30 | -6 ~ +6 | -10 | 0.81 | 0.81 | 0.81 | 0.81 |
| Amidation | Asparagine | 77 | -6 ~ +6 | -5 | 1.00 | 1.00 | 1.00 | 1.00 |
|  | Glycine | 143 | -6 ~ +6 | -5 | 1.00 | 0.96 | 1.00 | 0.98 |
|  | Isoleucine | 72 | -6 ~ +6 | -4 | 0.92 | 0.85 | 0.92 | 0.88 |
|  | Leucine | 263 | -6 ~ +6 | -4 | 0.92 | 0.92 | 0.92 | 0.92 |
|  | Methionine | 88 | -6 ~ +6 | -8 | 1.00 | 1.00 | 1.00 | 1.00 |
|  | Phenylalanine | 433 | -6 ~ +6 | -1 | 0.97 | 0.99 | 0.97 | 0.98 |
|  | Proline | 95 | -6 ~ +6 | -7 | 0.96 | 1.00 | 0.96 | 0.98 |
|  | Tyrosine | 88 | -6 ~ +6 | -7 | 1.00 | 1.00 | 1.00 | 1.00 |
| Sulfation | Tyrosine | 162 | -6 ~ +6 | -4.5 | 0.96 | 0.91 | 0.96 | 0.94 |
| Sumoylation | Lysine | 77 | -6 ~ +6 | -5 | 0.86 | 0.75 | 0.88 | 0.81 |
| Ubiquitination | Lysine | 284 | -6 ~ +6 | -5 | 0.82 | 0.67 | 0.85 | 0.76 |
| Pyrrolidone Carboxylic Acid | **Glutamate acid** | 598 | 0 ~ +6 | -4 | 0.76 | 0.69 | 0.79 | 0.74 |
| Gamma-Carboxyglutamic Acid | Glutamate | 371 | -6 ~ +6 | -4 | 0.92 | 0.90 | 0.93 | 0.91 |
| Nitration | Tyrosine | 47 | -6 ~ +6 | -3 | 0.85 | 0.65 | 0.81 | 0.73 |
| S-diacylglycerol cysteine | Cysteine | 36 | -6 ~ +6 | -5 | 1.00 | 0.94 | 1.00 | 0.97 |
| **Average** | |  |  |  | **0.87** | **0.82** | **0.86** | **0.84** |

**Table S3**. The list of integrated databases and programs.

| **Integrated Databases** | | |
| --- | --- | --- |
| **Database Name** | **Description** | **Statistics** |
| UniProtKB/Swiss-Prot [13, 14] | Protein variants | 32,101 variants corresponding to 6,115 proteins |
| RESID [15] | Annotations of Post-Translational Modification (PTM) | 431 PTM annotations |
| InterPro [16] | Protein domain | 1,113,928 entries can be corresponded to 247,238 Swiss-Prot entries |
| Protein Data Bank [17] | Protein structures | 30,937 entries can be corresponded to 10,274 Swiss-Prot proteins |
| COG [4] | Clusters of orthologous groups of proteins | 138,458 proteins form 4873 COGs in 66 genomes of unicellular organisms. The eukaryotic orthologous groups (KOGs) include proteins from 7 eukaryotic genomes consisting of 4852 clusters of orthologs, which include 59,838 proteins. |
| **Integrated Programs** | | |
| **Program Name** | **Description** | **Version** |
| KinasePhos **[18]** | Identifying Kinase-specific phosphorylation sites | Release 1.0 |
| DSSP [19] | Calculating the secondary structure and solvent accessibility of residues | April 1,2000 |
| RVP-net [20] | Predicting the solvent accessibility of residues | Release 1.0 |
| PSIPRED [21] | Predicting the protein secondary structures | Release 2.45 |
| Jmol[[1]](#footnote-2) | An open-source Java viewer for chemical structures in 3D | Release 11.2.4 |
| Weblogo [22] | Generating sequence logo for PTM substrates | Release 2.8.2 |
| Blast [6] | The programs BLASTCLUST and BL2SEQ were used to remove the redundant PTM sites | Release 2.2.12 |
| ClustalW [5] | Multiple sequences alignment in orthologous protein clusters | Release 1.83 |

**REFERENCE**

1. Mishra, G.R., et al., *Human protein reference database--2006 update.* Nucleic Acids Res, 2006. **34**(Database issue): p. D411-4.

2. Burge, C. and S. Karlin, *Prediction of complete gene structures in human genomic DNA.* J Mol Biol, 1997. **268**(1): p. 78-94.

3. Eddy, S.R., *Profile hidden Markov models.* Bioinformatics, 1998. **14**(9): p. 755-63.

4. Tatusov, R.L., et al., *The COG database: an updated version includes eukaryotes.* BMC Bioinformatics, 2003. **4**: p. 41.

5. Thompson, J.D., D.G. Higgins, and T.J. Gibson, *CLUSTAL W: improving the sensitivity of progressive multiple sequence alignment through sequence weighting, position-specific gap penalties and weight matrix choice.* Nucleic Acids Res, 1994. **22**(22): p. 4673-80.

6. Altschul, S.F., et al., *Gapped BLAST and PSI-BLAST: a new generation of protein database search programs.* Nucleic Acids Res, 1997. **25**(17): p. 3389-402.

7. Farriol-Mathis, N., et al., *Annotation of post-translational modifications in the Swiss-Prot knowledge base.* Proteomics, 2004. **4**(6): p. 1537-50.

8. Diella, F., et al., *Phospho.ELM: a database of phosphorylation sites--update 2008.* Nucleic Acids Res, 2008. **36**(Database issue): p. D240-4.

9. Gupta, R., et al., *O-GLYCBASE version 4.0: a revised database of O-glycosylated proteins.* Nucleic Acids Res, 1999. **27**(1): p. 370-2.

10. Chernorudskiy, A.L., et al., *UbiProt: a database of ubiquitylated proteins.* BMC Bioinformatics, 2007. **8**: p. 126.

11. Gnad, F., et al., *PHOSIDA (phosphorylation site database): management, structural and evolutionary investigation, and prediction of phosphosites.* Genome Biol, 2007. **8**(11): p. R250.

12. Diella, F., et al., *Phospho.ELM: a database of experimentally verified phosphorylation sites in eukaryotic proteins.* BMC Bioinformatics, 2004. **5**(1): p. 79.

13. Yip, Y.L., et al., *The Swiss-Prot variant page and the ModSNP database: a resource for sequence and structure information on human protein variants.* Hum Mutat, 2004. **23**(5): p. 464-70.

14. Boeckmann, B., et al., *The SWISS-PROT protein knowledgebase and its supplement TrEMBL in 2003.* Nucleic Acids Res, 2003. **31**(1): p. 365-70.

15. Garavelli, J.S., *The RESID Database of Protein Modifications as a resource and annotation tool.* Proteomics, 2004. **4**(6): p. 1527-33.

16. Mulder, N.J., et al., *InterPro: an integrated documentation resource for protein families, domains and functional sites.* Brief Bioinform, 2002. **3**(3): p. 225-35.

17. Deshpande, N., et al., *The RCSB Protein Data Bank: a redesigned query system and relational database based on the mmCIF schema.* Nucleic Acids Res, 2005. **33**(Database issue): p. D233-7.

18. Huang, H.D., et al., *KinasePhos: a web tool for identifying protein kinase-specific phosphorylation sites.* Nucleic Acids Res, 2005. **33**(Web Server issue): p. W226-9.

19. Kabsch, W. and C. Sander, *Dictionary of protein secondary structure: pattern recognition of hydrogen-bonded and geometrical features.* Biopolymers, 1983. **22**(12): p. 2577-637.

20. Ahmad, S., M.M. Gromiha, and A. Sarai, *RVP-net: online prediction of real valued accessible surface area of proteins from single sequences.* Bioinformatics, 2003. **19**(14): p. 1849-51.

21. McGuffin, L.J., K. Bryson, and D.T. Jones, *The PSIPRED protein structure prediction server.* Bioinformatics, 2000. **16**(4): p. 404-5.

22. Crooks, G.E., et al., *WebLogo: a sequence logo generator.* Genome Res, 2004. **14**(6): p. 1188-90.

1. Jmol: http://www.jmol.org/ [↑](#footnote-ref-2)
